# Safety of Bottle-Feeding Under Nasal Respiratory Support in Preterm Lambs With and Without Tachypnoea

**DOI:** 10.3389/fphys.2021.785086

**Published:** 2022-01-03

**Authors:** Basma Fathi Elsedawi, Nathalie Samson, Charlène Nadeau, Kristien Vanhaverbeke, Nam Nguyen, Charles Alain, Etienne Fortin-Pellerin, Jean-Paul Praud

**Affiliations:** ^1^Neonatal Respiratory Research Unit, Department of Pediatrics, Department of Physiology, University of Sherbrooke, Sherbrooke, QC, Canada; ^2^Department of Human Anatomy and Embryology, Faculty of Medicine, Zagazig University, Zagazig, Egypt; ^3^Laboratory of Experimental Medicine and Pediatrics, Infla-Med Centre of Excellence, University of Antwerp, Antwerp, Belgium; ^4^Department of Pediatrics, Antwerp University Hospital, Edegem, Belgium; ^5^Faculty of Human Medicine, Paracelsus Medical University, Nuremberg, Germany

**Keywords:** lamb, non-invasive respiratory support, oral feeding, preterm, sucking–swallowing–breathing coordination

## Abstract

**Aim:** Convalescing preterm infants often require non-invasive respiratory support, such as nasal continuous positive airway pressure or high-flow nasal cannulas. One challenging milestone for preterm infants is achieving full oral feeding. Some teams fear nasal respiratory support might disrupt sucking–swallowing–breathing coordination and induce severe cardiorespiratory events. The main objective of this study was to assess the safety of oral feeding of preterm lambs on nasal respiratory support, with or without tachypnoea.

**Methods:** Sucking, swallowing and breathing functions, as well as electrocardiogram, oxygen haemoglobin saturation, arterial blood gases and videofluoroscopic swallowing study were recorded in 15 preterm lambs during bottle-feeding. Four randomly ordered conditions were studied: control, nasal continuous positive airway pressure (6 cmH_2_O), high-flow nasal cannulas (7 L•min^–1^), and high-flow nasal cannulas at 7 L•min^–1^ at a tracheal pressure of 6 cmH_2_O. The recordings were repeated on days 7–8 and 13–14 to assess the effect of maturation.

**Results:** None of the respiratory support impaired the safety or efficiency of oral feeding, even with tachypnoea. No respiratory support systematically impacted sucking–swallowing–breathing coordination, with or without tachypnoea. No effect of maturation was found.

**Conclusion:** This translational physiology study, uniquely conducted in a relevant animal model of preterm infant with respiratory impairment, shows that nasal respiratory support does not impact the safety or efficiency of bottle-feeding or sucking–swallowing–breathing coordination. These results suggest that clinical studies on bottle-feeding in preterm infants under nasal continuous positive airway pressure and/or high-flow nasal cannulas can be safely undertaken.

## Introduction

One criterion commonly used worldwide for discharging preterm infants from the neonatal care unit is their ability to achieve safe and efficient full oral feeding (American Academy of Pediatrics Committee on Fetus and Newborn, 2008). Any delay in achieving this crucial physiological function will delay discharge from the neonatal intensive care unit and might result in growth failure, oral aversion, and poorer neurodevelopmental outcomes (Park et al., 2015; Jadcherla et al., 2017; Walsh et al., 2017; Hatch et al., 2018; Lainwala et al., 2020).

Nasal continuous positive airway pressure (nCPAP) and/or high-flow nasal cannulas (HFNCs) are commonly used in convalescing preterm infants to support their persistently impaired respiratory function non-invasively (Lemyre et al., 2016, 2017; Mahajan et al., 2016). These two modes of nasal respiratory support (NRS) act via somewhat different physiological mechanisms. Indeed, while nCPAP distends the upper airways and increases lung volumes by delivering a set level of positive pressure, the high flow rate of gas used with HFNCs instead washes the upper airways. High-flow nasal cannulas may—or may not—also provide an unknown level of distending positive pressure, depending on the infant’s weight, the gas flow rate, and the ratio between the diameters of the cannulas and the nares (Nasef et al., 2015).

Accordingly, oral feeding introduction under NRS is a highly controversial and debated topic amongst neonatologists due to the fear that NRS might further disrupt the physiologically immature sucking–swallowing–breathing (SU–SW–BR) coordination. As a result, it might induce harmful cardiorespiratory reflexes triggered by laryngeal penetration and/or tracheal aspiration (Thach, 2008; Boudaa et al., 2013). Consequently, oral feeding strategies in neonatal care units are greatly variable. Although some teams advocate that controlled introduction of oral feeding is safe under NRS in premature infants (Bonner and Mainous, 2008; Maastrup et al., 2012; Hanin et al., 2015; Glackin et al., 2017; Hasan et al., 2020), others strictly wait for the infant to be weaned from NRS before any oral feeding attempt (Nyqvist, 2008; Ferrara et al., 2017; Dumpa et al., 2020).

The persisting controversy on the safety of oral feeding under NRS led us to undertake a physiology translational research programme in newborn lambs to gain more extensive physiological knowledge on the effect of NRS on oral feeding. Our previous studies showed that bottle-feeding is safe under nCPAP in both healthy (Bernier et al., 2012) and tachypnoeic (Alain et al., 2021) full-term lambs, and in healthy preterm lambs (Samson et al., 2017). Similar results were obtained under HFNC in healthy (Samson et al., 2018), as well as tachypnoeic (Alain et al., 2021), full-term lambs. While informative and reassuring, the results yielded by these studies were, however, obtained in conditions at variance with convalescing preterm infants on NRS for respiratory impairment.

The main objective of this study was therefore to assess and compare the safety of bottle-feeding under nCPAP at 6 cmH_2_O and HFNC at 7 L•min^–1^ in our unique preterm lamb model, with and without tachypnoea. Our secondary objective was to assess and compare SU–SW–BR coordination, as well as the efficiency of bottle-feeding under the same conditions. We simultaneously tested the hypothesis that the presence of positive airway pressure might increase the efficiency of bottle-feeding, as suggested in a previous study (Samson et al., 2017). Lastly, we investigated the effect of postnatal maturation on the safety, SU–SW–BR coordination, and efficiency of bottle-feeding under NRS.

## Results

The study involved 15 preterm lambs (11 males) from six pregnant ewes. Five ewes gave birth to triplets, and one ewe gave birth to twins. Two of the 17 lambs died just after birth from dystocia or neonatal respiratory distress (survival rate of 88%). In addition, three lambs from the same litter died before postnatal days 13–14 due to pneumonia; their mother died a few days later, strongly suggesting their death was due to a communicable infectious disease. The mean weight of the 15 studied lambs was 2.7 ± 0.3 kg (min. 2.2 and max. 3.1 kg) at birth, and 3.5 ± 0.4 kg (min. 3.0 and max. 4.1 kg) and 4.5 ± 0.5 kg (min. 3.8 and max. 5.2 kg) on postnatal days 7 and 13, respectively.

Whatever the respiratory support, the presence or absence of tachypnoea, or postnatal age, it often took the preterm lamb several attempts to drink the whole 40 mL bottle (see Supplementary Table S1). As no meaningful differences were observed between attempts, the results were analysed and are presented with all attempts combined.

### Impact of Nasal Respiratory Support on Oral Feeding Without Tachypnoea

#### Safety of Bottle-Feeding

**FIGURE 1:**
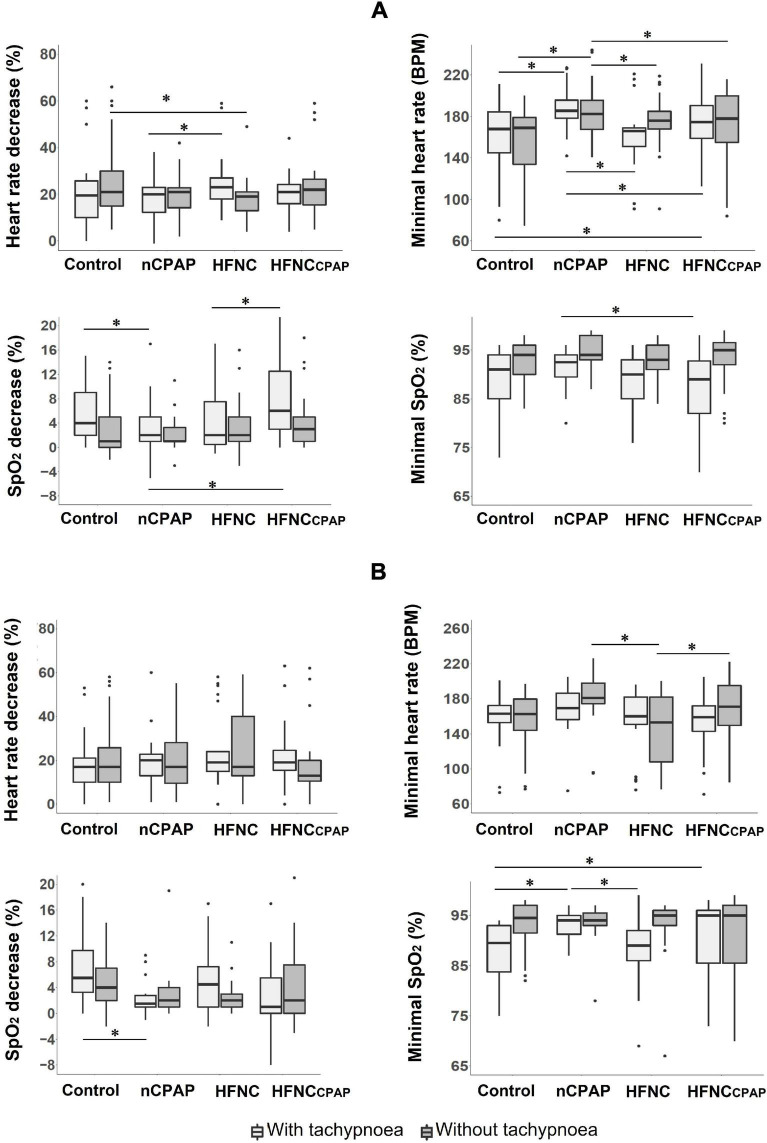
Safety of bottle-feeding in the three nasal respiratory support conditions studied. Four variables are displayed for each nasal respiratory support to illustrate the absence of significant cardiorespiratory events, with (light grey boxes) and without (dark grey boxes) tachypnoea, on postnatal days 7–8 **(A)** and 13–14 **(B)** recordings. Results are presented as median (Q1, Q3); * indicates a statistically significant difference (*p* < 0.05). For recordings on postnatal days 7–8, nCPAP (*n* = 14 with and *n* = 15 without tachypnoea) and HFNCcpap (*n* = 14 with and *n* = 15 without tachypnoea) significantly increased minimal HR compared to control (*n* = 15 with and without tachypnoea), whereas the percentage of HR decrease was lower with HFNC (*n* = 13 with and *n* = 14 without tachypnoea). For recordings on postnatal days 13–14, a lower minimal heart rate was observed with HFNC (*n* = 11 with and without tachypnoea) when compared to nCPAP (*n* = 9 with and *n* = 11 without tachypnoea) and HFNCcpap (*n* = 11 with and without tachypnoea) in the absence of tachypnoea. For postnatal days 13–14, *n* = 12 with and without tachypnoea in the control condition. CTRL, control condition (no respiratory support); nCPAP, nasal continuous positive airway pressure at 6 cmH_2_O; HFNC, high-flow nasal cannula at 7 L•min^–1^; HFNCcpap, high-flow nasal cannula at 7 L•min^–1^ with end-expiratory tracheal pressure equivalent to nCPAP. The scattered dots represent outlier data points which were included in the analysis.

None of the three NRS conditions negatively impacted the safety variables—heart rate, respiratory rate, transcutaneous oxygen haemoglobin saturation (SpO_2_), arterial blood gases—during bottle-feeding on postnatal days 7–8 (Figure 1A and Supplementary Table S2). In contrast, nCPAP significantly increased minimal HR compared to control, whereas the percentage of HR decrease was reduced with HFNC. Analysis of videofluoroscopy revealed no laryngeal penetration nor tracheal aspiration during any of the experimental conditions. Of note, however, the preterm lambs often had difficulty latching onto the teat—especially in the nCPAP condition with the nasal mask in place—so that an experimenter had to assist them. This prevented conducting the videofluoroscopy assessment in up to 67% of the cases with nCPAP (see Supplementary Table S1). Lastly, none of the NRS conditions negatively impacted blood gases at baseline or 1 min after feeding (Supplementary Table S3).

Similarly, none of the three NRS conditions significantly impacted the safety variables during bottle-feeding on postnatal days 13–14 (Supplementary Figure S1 and Supplementary Table S4). The only statistically significant difference was a lower minimal heart rate with HFNC when compared to nCPAP and HFNCcpap. Of note, all of the three types of NRS significantly increased PaO_2_ compared to the control condition (Supplementary Table S5).

#### Sucking–Swallowing–Breathing Coordination

**FIGURE 2:**
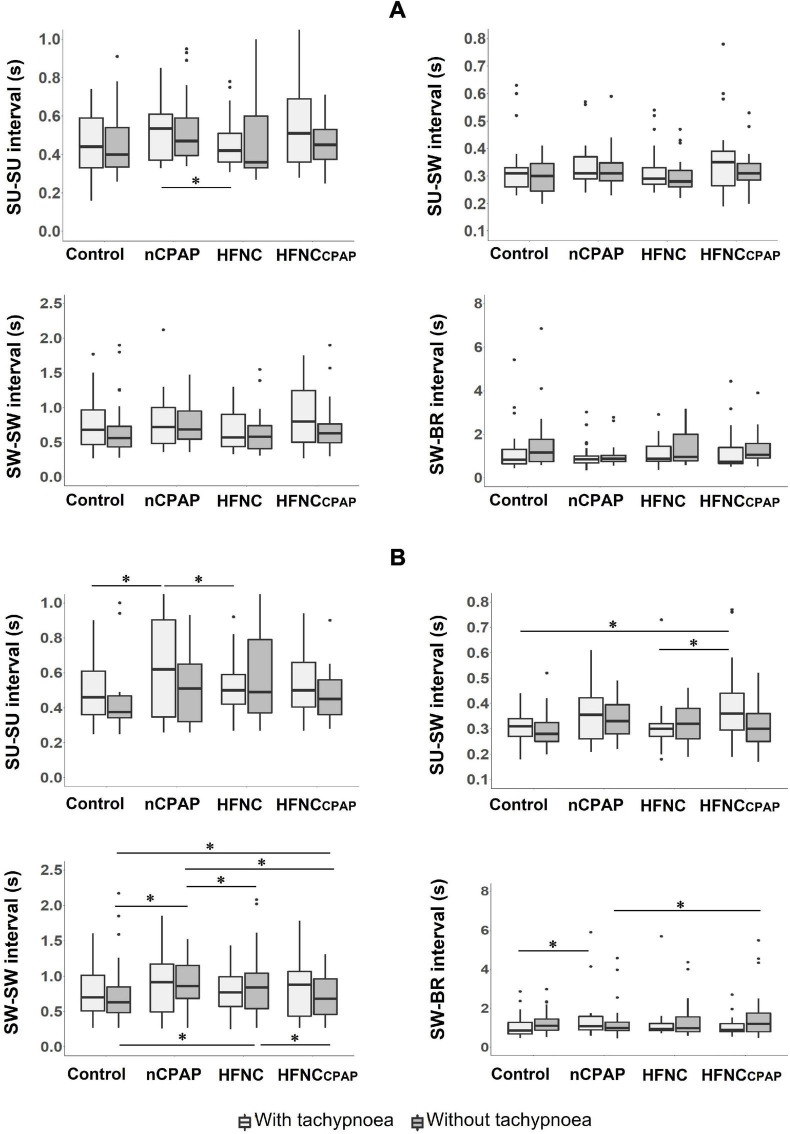
Sucking (SU)–swallowing (SW)–breathing (BR) coordination during bottle-feeding in the three nasal respiratory support conditions studied. Four variables are displayed for each nasal respiratory support to illustrate sucking–swallowing–breathing coordination, with (light grey boxes) and without (dark grey boxes) tachypnoea, on postnatal days 7–8 **(A)** and 13–14 **(B)** recordings. Of note, no alterations of SU–SW–BR coordination were observed on postnatal days 7–8. Results are presented as median (Q1, Q3); * indicates a statistically significant difference (*p* < 0.05). For postnatal days 7–8, control (*n* = 15 with and without tachypnoea), nCPAP (*n* = 14 with and *n* = 15 without tachypnoea), HFNC (*n* = 13 with and *n* = 14 without tachypnoea), and HFNCcpap (*n* = 14 with and *n* = 15 without tachypnoea). For postnatal days 13–14, control (*n* = 12 with and without tachypnoea), nCPAP (*n* = 9 with and *n* = 11 without tachypnoea), HFNC (*n* = 11 with and without tachypnoea), and HFNCcpap (*n* = 11 with and without tachypnoea). Please see Figure 1 for abbreviations. The scattered dots represent outlier data points which were included in the analysis.

No alterations in SU–SW–BR coordination were observed during recordings on days 7–8 for any of the three NRS conditions tested, apart from a significant increase in SW–BR COV with HFNCcpap compared to the control condition (Figure 2A and Supplementary Table S2). On days 13–14, however, nCPAP, HFNC, and HFNCcpap increased the SW–SW interval, and nCPAP significantly decreased SU–SU COV compared to the control condition and HFNCcpap (Figure 2B and Supplementary Table S4).

#### Efficiency of Bottle-Feeding

**FIGURE 3:**
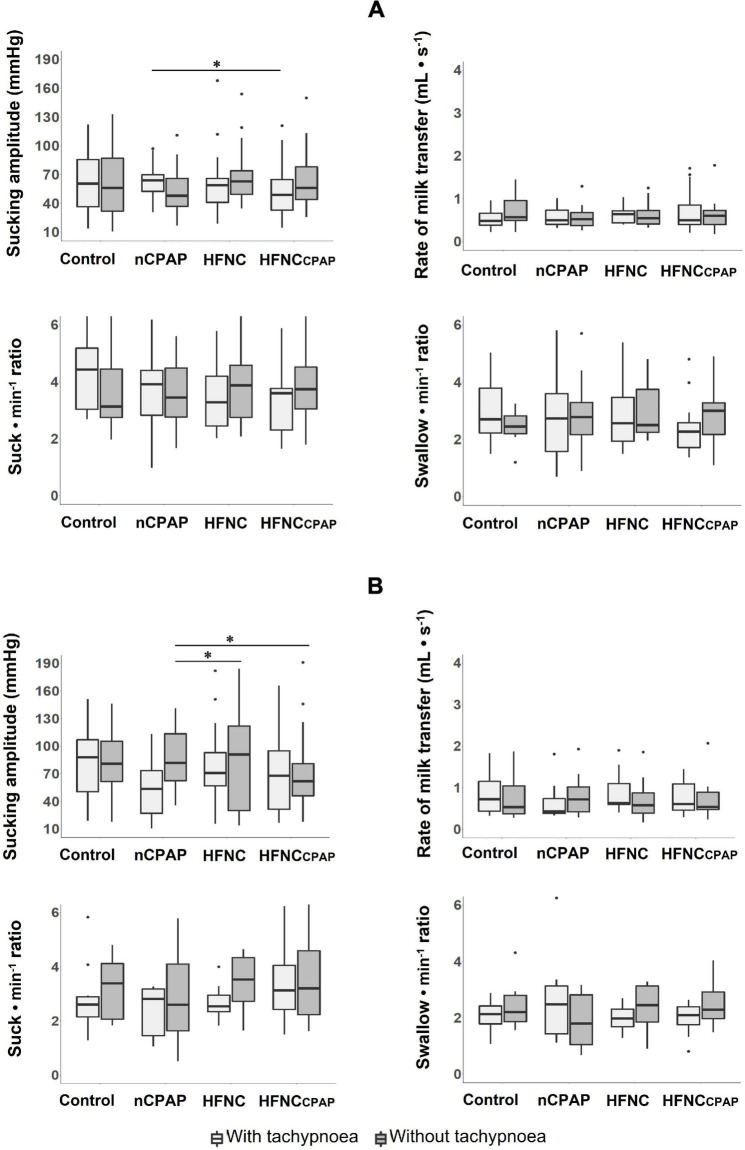
Efficiency of oral feeding in the three nasal respiratory support conditions studied. Four variables are displayed for each nasal respiratory support to illustrate feeding efficiency, with (light grey boxes) and without (dark grey boxes) tachypnoea, on postnatal days 7–8 **(A)** and 13–14 **(B)** recordings. Of note, no efficiency indices were modified by any of the NRS conditions tested on postnatal days 7–8. Results are presented as median (Q1 and Q3); * indicates a statistically significant difference (*p* < 0.05). See Figure 2 legend for absolute numbers of lambs studied in each experimental condition, and Figure 1 for abbreviations. The scattered dots represent outlier data points which were included in the analysis.

Compared to the control condition, none of the three NRSs tested altered any indices of efficiency on days 7–8 and days 13–14 (Figure 3A and Supplementary Tables S2, S4). Sucking amplitude with nCPAP was greater than with HFNCcpap, but it was lower when compared to HFNC on days 13–14 (Figure 3B and Supplementary Table S4).

### Impact of Imposed Tachypnoea on Bottle-Feeding

Standardised tachypnoea—50% increase in the baseline respiratory rate—was successfully achieved in all preterm lambs under all the conditions (Supplementary Table S6). Apart from tachypnoea, close observation did not reveal any other clinical signs of respiratory distress in any animal.

On postnatal days 7–8, despite tachypnoea, none of the three NRS conditions negatively impacted the safety variables during bottle-feeding (Figure 1A and Supplementary Table S7). On the contrary, minimal HR and minimal SpO_2_ were greater with nCPAP than the other conditions. None of the NRS conditions negatively impacted arterial blood gases and pH at baseline or 1 min after feeding (Supplementary Table S8). Analysis of videofluoroscopy did not reveal any laryngeal penetration or tracheal aspiration. Again, videofluoroscopic assessment often could not be performed due to the need for the experimenter’s assistance, especially with nCPAP (see Supplementary Table S1). As for SU–SW–BR coordination, SU–SU interval was greater with nCPAP than with HFNC; SU–SU COV was lower with HFNC than with the other three experimental conditions (Figure 2A and Supplementary Table S7). Lastly, efficiency of bottle-feeding was not altered by any NRS condition (Figure 3A and Supplementary Table S7).

On postnatal days 13–14, none of the three NRS conditions significantly impacted the safety and efficiency of bottle-feeding or the SU–SW–BR coordination (Figures 1B, 2B, 3B). Laryngeal penetration or tracheal aspiration was never observed when videofluoroscopic assessment could be conducted (see Supplementary Table S1). Compared to the control condition, SU–SU and SW–BR intervals were higher with nCPAP, and SU–SW interval was higher with HFNCcpap (Supplementary Table S9). In addition, apart from a slightly lower PaO_2_ with HFNC and HFNCcpap compared to control condition at baseline, none of the NRS conditions negatively impacted blood gases before or 1 min after feeding (Supplementary Table S10).

Compared to experiments without tachypnoea, the SW–SW interval, as well as arterial CO_2_ at 1 min after bottle-feeding was significantly higher on postnatal days 7–8 (Supplementary Tables S11, S12). In contrast, the following variables were significantly lower: baseline SpO_2_, minimal SpO_2_, SpO_2_ decrease, arterial O_2_ pressure both at baseline and 1 min after bottle-feeding, arterial pH at 1 min after feeding, as well as the SW–BR interval and SW–BR COV.

In addition, on postnatal days 13–14, mean SpO_2_, minimal SpO_2_, and arterial O_2_ pressure were lower at baseline and 1 min after bottle-feeding (Supplementary Tables S11, S12).

## Discussion

This study conducted in our preterm lamb model uniquely and importantly shows that nCPAP 6 cmH_2_O, HFNC 7 L•min^–1^, and HFNCcpap do not significantly impair the safety of oral feeding, even in the presence of tachypnoea. This includes the absence of laryngeal penetration or tracheal aspiration during any of the experimental conditions. Moreover, bottle-feeding efficiency remained unaltered with all of the tested NRSs. Lastly, none of the NRSs modified SU–SW–BR coordination in a systematic way.

### The Continuing Debate on Bottle-Feeding During Nasal Respiratory Support and the Relevance to Conduct Translational Physiology Studies in an Appropriate Animal Model

In infants born prematurely, the maturation of SU–SW–BR coordination commonly allows for initiating oral feeding around 32–34 weeks post-gestational age (Mizuno and Ueda, 2003; Medhurst, 2005; Breton and Steinwender, 2008). At that time, however, the convalescent preterm infant often requires some form of NRS. As a result, caregivers are faced with the unresolved conundrum of risking either cardiorespiratory events during attempts at oral feeding under NRS or the consequences of delaying oral feeding. A recent survey of practice of NICUs and PICUs in Australia and New Zealand reported that most units do not feed on NRS as aspiration risks are unclear (Canning et al., 2020). In addition, a recent systematic review of clinical studies concluded that there is insufficient evidence to recommend the introduction of oral feeding whilst on nCPAP or HFNC to facilitate transition to full oral feeding without adverse effects (Canning et al., 2021).

Given the strong caregiver opinions, the lack of evidence-based guidelines, and the consequent high variability in feeding practices under NRS, we believe that carefully planned preclinical studies in an appropriate animal model can provide the physiological knowledge required to design and conduct safer and more decisive clinical studies in human infants. Our research programme in newborn lambs allows us to assess the safety and the SU–SW–BR coordination more thoroughly in the same subject under different NRS conditions—including no NRS—and under repeated radiation exposure. Our current study represents our unique attempt at mimicking a tachypnoeic preterm infant at ∼34 weeks of postconceptional age.

### Safety of Oral Feeding While on Nasal Respiratory Support

This study importantly revealed that oral feeding is safe under nCPAP at 6 cmH_2_O, HFNC at 7 L•min^–1^, or HFNCcpap in preterm lambs 7–8 days old, as well as after reaching full-term postconceptional age, even in the presence of tachypnoea. Indeed, none of the cardiorespiratory variables assessed were negatively impacted by any of the tested NRSs. This absence of any significant cardiorespiratory inhibition under all tested experimental conditions is a strong argument against laryngeal penetrations. Indeed, past studies conducted by our team in the same preterm lamb model have shown that laryngeal penetrations triggered foetal-type laryngeal chemoreflexes characterised by bradycardia, apnoea, oxygen haemoglobin desaturation, and laryngospasm (St-Hilaire et al., 2007). In addition to the absence of cardiorespiratory events, videofluoroscopy assessment, when it could be performed, did not reveal any laryngeal penetration or tracheal aspiration regardless of the experimental condition. These results agree with past studies reporting no clinical signs suggestive of aspiration in convalescing preterm infants with bronchopulmonary dysplasia or orally fed while on nCPAP (Hanin et al., 2015; Dalgleish et al., 2016; Jadcherla et al., 2016; Glackin et al., 2017; Shimizu et al., 2019). The results are, however, contrary to one single videofluoroscopy study showing a significant increase in deep laryngeal penetration and aspiration episodes in preterm infants bottle-fed on nCPAP (Ferrara et al., 2017). Of note, our recent observations in full-term lambs revealed very rare laryngeal penetrations and tracheal aspirations occurring almost exclusively with HFNC, especially with HFNCcpap (Alain et al., 2021). The reason for this slight discrepancy between our two studies is unclear. It could, however, be partly due to the impossibility of obtaining videofluoroscopic assessment under all experimental conditions in preterm lambs. Nevertheless, pooled results obtained in newborn lambs—full-term and preterm—clearly show that laryngeal penetration and tracheal aspiration remain rare occurrences, with no deleterious cardiorespiratory consequences.

### Impact of Nasal Respiratory Support on Sucking–Swallowing–Breathing Coordination

We only found a few statistically significant differences supporting an impact of NRS on SU–SW–BR coordination. These differences, which seem to predominate on postnatal days 13–14, remain unexplained. The most notable results might be the increased SW–SW interval by all three NRSs in the absence of tachypnoea, as well as the increase in both SU–SU and SW–BR intervals under nCPAP in the presence of tachypnoea. These results are of uncertain physiological significance. Overall, we believe the two important messages are the following. First, none of the NRSs had a systematic effect on SU–SW–BR coordination. Second, whatever the NRS condition and the presence or not of tachypnoea, preterm lambs adopted an appropriate SU–SW–BR strategy to successfully feed orally without triggering deleterious cardiorespiratory reflexes.

One peculiar aspect of SU–SW–BR coordination in preterm subjects is worth discussing. It is well documented in both humans (Gewolb et al., 2001; Gewolb and Vice, 2006; Barlow, 2009) and animal models (Samson et al., 2017; Mayerl et al., 2019) that SU–SW–BR coordination is different in preterm and full-term infants. In particular, the percentage of nutritive swallows occurring during an apnoea is lower in full-term lambs (Samson et al., 2018) than in preterm ones (Samson et al., 2017), which is in agreement with results obtained in human infants (Lau et al., 2003; Mizuno and Ueda, 2003; Gewolb and Vice, 2006). This difference between preterm and full-term lambs, however, appears to hold true only when the bottle is held by an experimenter. Indeed, we recently observed that an important proportion—almost half—of nutritive swallows occurred during episodes of apnoea in full-term lambs also when they fed unassisted from our bottle-feeding device (Alain et al., 2021). This observation remains unexplained. The present results in preterm lambs unfortunately do not yield a clear picture of this aspect of SU–SW–BR coordination, for the latter might have been modified in the lamb needing assistance during multiple attempts at bottle-feeding.

### Impact of Nasal Respiratory Support on Oral Feeding Efficiency

In recordings obtained at days 7–8, no indices of bottle-feeding efficiency were modified by any of the NRSs tested. These results are in accordance with our most recent study in full-term lambs, which used a similar experimental design (Alain et al., 2021). The results herein, however, do not replicate those obtained previously in preterm and full-term lambs (Samson et al., 2017, 2018), which documented increased feeding efficiency under nCPAP compared to no NRS or to HFNC. We believe that the discrepancy between our studies is attributable to the specific study design required for videofluoroscopy assessment. Accordingly, during videofluoroscopy, lambs had to drink by themselves from our custom-made bottle-feeding device (Alain et al., 2021), conversely to our two initial studies in which the lambs were gently held by the same experimenter offering a bottle.

On days 13–14, sucking amplitude was greater during nCPAP than during HFNCcpap. This finding is somewhat in agreement with our recent study, which showed that HFNCcpap decreased feeding efficiency in full-term lambs (Alain et al., 2021).

### Limitations of Our Study

First, we acknowledge that the use of thoracic compression to induce tachypnoea does not reproduce the pathophysiology of any common respiratory disease in the preterm. The relevance of studying tachypnoea, however, stems from the fact that it is virtually always present in preterm infants with bronchopulmonary dysplasia. In addition, it is the only standardisable way we found to impair respiration transiently, which was a must for our study. Second, our videofluoroscopy equipment did not allow us to use a rate higher than 12 frames•s^–1^, which is slightly less than the recommended 15 frames•s^–1^ in children (Hiorns and Ryan, 2006; Layly et al., 2020). This may have prevented us from recognising some laryngeal penetrations or tracheal aspirations. Third, the prone position with an extended neck—naturally adopted by newborn lambs while bottle-feeding—clearly does not reproduce the feeding position of a human newborn. Fourth, given that all lambs had been suckled from birth by their mothers, they had certainly developed some sucking–swallowing–breathing coordination prior to the experiment. Consequently, our results were obtained in a setting different from preterm infants, whose oral feeding is initiated under respiratory support. Fifth, although initially included in the experimental design, the effect of sex unfortunately could not be assessed due to the low number of female lambs born (4 females vs. 11 males). Lastly, although we unequivocally acknowledge that results obtained in preterm lambs cannot be taken as fully representing data in human preterms, many anatomical and functional characteristics of nutritive swallowing are common to both species. Anatomically, the upper aerodigestive tract is divided into an airway and a foodway with an overlapping epiglottis and soft palate in both species. The ovine larynx has an overall similar structure and is used for human surgical training (Isaacson et al., 2015, 2016; Ianacone et al., 2016). In both species, the cricopharyngeus muscle is the main component of the upper oesophageal sphincter (Samson et al., 2021). Functionally, there is similar immaturity of swallowing–breathing coordination during nutritive swallowing (frequent apnoeas during oral feeding) (Lau et al., 2003; Mizuno and Ueda, 2003; Gewolb and Vice, 2006). Similar vagovagal laryngeal chemoreflexes characterised by cardiorespiratory events can be elicited (Pickens et al., 1988; St-Hilaire et al., 2007).

## Conclusion

Results from the present study show that bottle-feeding is safe in lambs born 14 days prematurely and studied at postnatal days 7–8 under nCPAP at 6 cmH_2_O, HFNC at 7 L•min^–1^, or HFNCcpap, even in the presence of an artificially induced transient tachypnoea. In addition, maturation until a postconceptional age identical to full-term does not modify the results. Lastly, feeding efficiency and sucking–swallowing–breathing coordination are not significantly altered by any of the three NRSs tested. Altogether, results from our preclinical experiments are reassuring and provide the foundation for performing clinical studies on bottle-feeding in convalescing preterm infants without *a priori* favouring nCPAP or HFNC.

## Materials and Methods

The study was carried out in accordance with the recommendations of the Canadian Council on Animal Care. The protocol was approved by the Ethics Committee for Animal Care and Experimentation of the Université de Sherbrooke (protocol #2018–2051). Experiments were conducted on 15 mixed-bred Dorset-Romanov preterm lambs (11 males) obtained from a local breeder and born 14 days prematurely as previously described (Boudaa et al., 2013). Briefly, premature labour was induced by mifepristone (8 mg•kg^–1^) after stimulation of lung maturation by intramuscular betamethasone (12 mg × 2). Vital signs, including body temperature, heart and respiratory rate, SpO_2_, blood glucose level, and weight, were regularly monitored up to the first recording day. This included the continuous presence of an experimenter during the first postnatal 48 h to detect and treat any hypothermia, hypoglycaemia, or oxygen haemoglobin desaturation. Preterm lambs stayed with and fed on their ewe; they weighed 3.5 ± 0.4 kg at day 7 of life. All lambs surviving the first postnatal hours were included *a priori* in the study. Exclusion criteria included the presence of any respiratory problem, infection, or any other significant health problem on the experimental days. The number of pregnant ewes was calculated *a priori* from our past experience with survival rate (∼80% on average) with this unique preterm lamb model. We also hoped to obtain about 7 preterm females and 7 preterm males to test for the effect of sex as a secondary objective, while restricting the total number of lambs for ethical reasons. The ewes were returned to the sheep farm at the end of the experiments and were eligible for subsequent gestations, as part of our research programme on preterm lambs, before being euthanised.

### Chronic Instrumentation and Recording Equipment

Details of the chronic instrumentation have been described elsewhere (Samson et al., 2017; Alain et al., 2021). Briefly, on postnatal day 6, chronic surgical instrumentation was performed under local anaesthesia and included the insertion of (1) custom-built bipolar electrodes into both thyroarytenoid (EAta, a laryngeal constrictor) muscles for recording of swallowing activity; (2) a catheter into the left carotid artery to measure arterial blood gases (RapidLab 348, Siemens, Saint-Laurent, Canada); and (3) a transcutaneous catheter between the fifth and sixth tracheal rings to monitor tracheal-pressure variations.

Further instrumentation of the lambs was completed immediately before recordings and included (1) subcutaneous needle electrodes for electrocardiogram (ECG) recordings; (2) thoracic and abdominal elastic bands to monitor lung volume variations semi-quantitatively via respiratory inductance plethysmography (Ambulatory Monitoring, Ardsley, NY, United States); and (3) a pulse oximetry probe (LNOP YI reflectance sensor, Masimo, Irvine, CA, United States) at the base of the tail for continuous monitoring of SpO_2_.

As previously described, nasal continuous positive airway pressure was delivered with the Infant Flow^®^ nCPAP system (Cardinal Health, Dublin, OH, United States), which delivered a variable-flow nCPAP of 6 cmH_2_O through a custom-designed plastic nasal mask for newborn lambs (Supplementary Figure S1). A high nasal flow of 7 L•min^–1^ of air was delivered with the Optiflow™ system (BC2755, Fisher & Paykel, Mississauga, ON, Canada) with small infant-size nasal cannulas. Lastly, given our past observation that bottle-feeding efficiency was enhanced under nCPAP (6 cmH_2_O) but not under HFNC (7 L•min^–1^) (Samson et al., 2018), we also studied a third type of NRS combining HFNC and a positive airway pressure of 6 cmH_2_O (HFNCcpap). The latter was achieved with the Optiflow™ system at 7 L•min^–1^ with small adult-size nasal cannulas and by reducing leaks at the nares with dental-impression material (EXAMIX^®^, Henry Schein Canada, ON, Canada) to obtain an end-expiratory tracheal pressure of 6 cmH_2_O (Alain et al., 2021). Lastly, for the control condition, the lambs were bottle-fed without any respiratory support and without a nasal mask. The air administered with all forms of NRS was heated and humidified. No oxygen was given to any of the lambs.

The lambs were fed without assistance from a custom-designed bottle-feeding device developed to allow videofluoroscopic studies without exposing the personnel to radiation (Supplementary Figure S2) (Alain et al., 2021). A saline-filled catheter connected to a pressure transducer (TSD104A pressure transducer, Biopac Systems Canada, Montreal, QC, Canada) was introduced in the teat of the bottle to record sucking activity (positive expression amplitude). The system allowed for adjusting teat height according to animal morphology.

All bottle-feeding sessions were performed under videofluoroscopy [Philips BV Pulsera C-Arm (Markham, ON, Canada)] to assess laryngeal penetration and/or tracheal aspiration. A mixture of 25% barium solution (Liquid Polibar Plus, E-Z-EM, Anjou, QC, Canada) and 75% milk was used to achieve a proper radiological contrast without modifying the texture, thickness, or colour of the milk (previously quantified in pilot experiments in 2 full-term lambs). The C-Arm was operated in sequential mode and set to 12 frames•s^–1^ with a fixed X-ray energy setting in the 49–53 kVp range and a current intensity of 0.4–0.8 mA, as required for contrast in a given subject.

Physiological signals were transmitted wirelessly with our custom-designed radiotelemetry system (Samson et al., 2011) and continuously recorded (AcqKnowledge software, version 4.1, Biopac Systems Canada, Montreal, QC, Canada). The entire recording period was video recorded with a webcam, and an experimenter was present to note all events occurring during the recordings.

### Design of the Study

Following their birth, all preterm lambs were cared for with their mother in our animal quarters and were able to feed *ad libitum* on the ewe. Bottle-feeding was initiated on postnatal days 7–8 and repeated on days 13–14 to study the effect of maturation. The lambs were comfortably positioned prone in a sling with loose restraint. On the day of the recordings, one bottle of 40 mL of ewe milk—i.e., the maximum amount of milk that preterm lambs were able to drink during one bottle-feeding attempt, as determined in pilot experiments—was offered to each animal in the control condition (no NRS) and each of the 3 NRS conditions in random order (block randomisation) one day with and one day without respiratory impairment (randomly ordered days) under videofluoroscopic examination. A 3-h interval was systematically respected between two bottle-feedings (Alain et al., 2021). Videofluoroscopic recordings were continued for 20 s after bottle-feedings. The experimental conditions included no respiratory support (control), nCPAP at 6 cmH_2_O, HFNC at 7 L•min^–1^, and HFNCcpap. Respiratory impairment (tachypnoea) was induced by inflating a blood-pressure cuff around the thorax and the upper abdomen until the respiratory rate increased by 50%.

### Data Analysis

As extensively detailed previously (Samson et al., 2017, 2018; Alain et al., 2021), all physiological signals were analysed to quantify the safety and efficiency of bottle-feeding, as well as the SU–SW–BR coordination (rhythmic stability of feeding) of each bottle-feeding attempt in each of the three NRS and the control conditions, with and without tachypnoea. The data was analysed 20 s before bottle-feeding (=baseline), throughout all the bottle-feeding attempts and subsequently during 30 s.

The safety of bottle-feeding was quantified by computing the following variables: the number of heart-rate (HR) decelerations (defined by a percentage decrease in HR of 33% for a maximum of 5 s) and bradycardias (HR slowing >5 s), the minimal HR (min^–1^), the total duration of cardiac inhibition (sum of all the HR decelerations and bradycardias for each reflex in seconds), the percentage decrease in HR (decrease in percentage from baseline HR to minimal HR during bottle-feeding), the minimal SpO_2_ (%), the percentage decrease in SpO_2_ (%), the number of coughs (defined by a distinctive audible sound combined with a brisk and short increase in tracheal pressure and EAta), the number of laryngeal penetrations (presence of milk in the laryngeal vestibule above the glottis), and tracheal aspirations (presence of milk below the glottis) (Ferrara et al., 2017). The videofluoroscopic recordings were analysed with a RadiAnt DICOM Viewer (version 5.0.0, Poznań, Poland)^1^.

The rhythmic stability of feeding was quantified by computing the time interval between two sucks (SU–SU) and between two swallows (SW–SW), as well as the SU–SW and SW–breath (BR) intervals. The coefficients of variation [COV = standard deviation of the mean interval divided by the mean interval (Gewolb et al., 2001)] of SU–SU, SU–SW, SW–SW, and SW–BR intervals were also calculated. A lower COV value indicates a more stable rhythm (Da Costa et al., 2008). In addition, the percentage of feeding duration spent in apnoea (defined as at least two missed breaths with an amplitude 30% lower than baseline breathing (Samson et al., 2017) as well as the percentage of swallows occurring during an apnoea, was calculated.

The efficiency of bottle-feeding was quantified by computing the rate of milk transfer (mL•s^–1^), the number of sucks (sucks•mL^–1^) and swallows (swallows•mL^–1^) needed to drink one mL of milk, and the mean positive pressure in the teat.

End-expiratory tracheal pressure was used to measure the positive pressure applied to the respiratory system by nCPAP, HFNC, and HFNCcpap (Supplementary Table S13). Lastly, the respiratory rate (RR), HR, mean SpO_2_, and arterial blood gases (PaCO_2_, PaO_2_, and pH) were measured just before and 1 min after bottle-feeding.

### Statistical Analysis

All statistical analyses were performed with R V. 3.6.0 (The R Project for statistical computing, 2019, Vienna, Austria), with the help of the Department of Biostatistics of our research centre. A total of 37 dependent variables were evaluated. We first compared the impact of the three NRS conditions on the safety of bottle-feeding with and without tachypnoea. Similar comparisons were performed for efficiency and SU–SW–BR coordination. All analyses were initially conducted on the bottle-feeding periods without tachypnoea only and repeated for the bottle-feeding periods with tachypnoea. For each dependent variable, a generalised linear mixed-model analysis was performed. Tukey’s *post hoc* analysis was used for multiple comparisons of the mean differences between the conditions (a *p*-value < 0.05 was deemed significant). For each variable, the most representative distribution was selected based on the Akaike information criterion. Gamma, lognormal, or normal distributions were used for continuous variables, while Bayesian distribution was always the most appropriate one for discrete variables. The effect of maturation was tested using the same statistical model. Results are presented as median and first and third quartiles (Q1 and Q3).

## Data Availability Statement

The original contributions presented in the study are included in the article/Supplementary Material, further inquiries can be directed to the corresponding author/s.

## Ethics Statement

The animal study was reviewed and approved by Ethics Committee for Animal Care and Experimentation of the Université de Sherbrooke. Written informed consent was obtained from the owners for the participation of their animals in this study.

## Author Contributions

NS, CN, and J-PP conceived and designed the research. NS and CN performed the deliveries. BFE, NS, CN, KV and NN ensured the care of the preterm lambs. BFE, NS, and CN performed the animal experiments. BFE, NS, CN, and CA analysed the data. BFE, NS, and J-PP interpreted the results and wrote the manuscript. BFE, NS, and CA prepared the figures. All authors read and approved the final version of the manuscript.

## Conflict of Interest

The authors declare that the research was conducted in the absence of any commercial or financial relationships that could be construed as a potential conflict of interest.

## Publisher’s Note

All claims expressed in this article are solely those of the authors and do not necessarily represent those of their affiliated organizations, or those of the publisher, the editors and the reviewers. Any product that may be evaluated in this article, or claim that may be made by its manufacturer, is not guaranteed or endorsed by the publisher.

## Footnotes

1
https://www.radiantviewer.com/

## Abbreviations

nCPAP: nasal continuous positive airway pressure
HFNC: high-flow nasal cannula
HFNCcpap: high-flow nasal cannula with an end-expiratory tracheal pressure of 6 cmH_2_O
CTRL: control condition
NRS: nasal respiratory support
SU–SW–BR: sucking–swallowing–breathing
COV: coefficient of variation
Q1: first quartile
Q3: third quartile
HR: heart rate
RR: respiratory rate
SpO_2_: oxygen saturation
EAta: thyroarytenoid electrodes
ECG: electrocardiogram
PaO_2_: arterial oxygen pressure
PaCO_2_: arterial CO_2_ pressure.

## References

[B1] Alain C. Samson N. Nadeau C. Beaudoin J. F. Lienhart C. Counil C. (2021). Nasal respiratory support and tachypnea and oral feeding in full-term newborn lambs. *J. Appl. Physiol.* 130 1436–1447. 10.1152/japplphysiol.00567.2020 33661723

[B2] American Academy of Pediatrics Committee on Fetus and Newborn (2008). Hospital discharge of the high-risk neonate. *Pediatrics* 122 1119–1126. 18977994 10.1542/peds.2008-2174

[B3] Barlow S. M. (2009). Oral and respiratory control for preterm feeding. *Curr. Opin. Otolaryngol. Head Neck Surg.* 17 179–186. 10.1097/MOO.0b013e32832b36fe 19369871 PMC2724868

[B4] Bernier A. Catelin C. Ahmed M. A. H. Samson N. Bonneau P. Praud J. P. (2012). Effects of nasal continuous positive-airway pressure on nutritive swallowing in lambs. *J. Appl. Physiol.* 112 1984–1991. 10.1152/japplphysiol.01559.2011 22500003

[B5] Bonner K. M. Mainous R. O. (2008). The nursing care of infant receiving bubble CPAP therapy. *Adv. Neonat. Care* 8 78–95. 10.1097/01.ANC.0000317256.76201.72 18418205

[B6] Boudaa N. Samson N. Carrière V. Germim P. S. Pasquier J. C. Bairam A. (2013). Effects of caffeine and/or nasal CPAP treatment on laryngeal chemoreflexes in preterm lambs. *J. Appl. Physiol.* 114 637–646. 10.1152/japplphysiol.00599.2012 23305977

[B7] Breton S. Steinwender S. (2008). Timing introduction and transition to oral feeding in preterm infants; current trends and practice. *Newborn Infant. Nurs. Rev.* 8 153–159.

[B8] Canning A. Clarke S. Thorning S. Chauhan M. Weir K. A. (2021). Oral feeding for infants and children receiving nasal continuous positive airway pressure and high flow nasal cannula: a systematic review. *BMC Pediatr.* 21:83. 10.1186/s12887-021-02531-4 33596866 PMC7887825

[B9] Canning A. Fairhurst R. Chauhan M. Weir K. A. (2020). Oral feeding for infants and children receiving nasal continuous positive airway pressure and high-flow nasal cannula respiratory supports: a survey of practice. *Dysphagia* 35 443–454. 10.1007/s00455-019-10047-4 31451906

[B10] Da Costa S. P. Van Den Engel-Hoek L. Bos A. F. (2008). Sucking and swallowing in infants and diagnostic tools. *J. Perinatol.* 28 247–257. 10.1038/sj.jp.7211924 18200022

[B11] Dalgleish S. R. Kostecky L. L. Blachly N. (2016). Eating in “SINC”: safe individualized nipple-feeding competence, a quality improvement project to explore infant driven oral feeding for very premature infants requiring noninvasive respiratory support. *Neonatal Netw.* 35 217–227. 10.1891/0730-0832.35.4.217 27461200

[B12] Dumpa V. Kamity R. Ferrara L. Akerman N. Hanna N. (2020). The effects of oral feeding while on nasal continuous positive airway pressure (NCPAP) in preterm infants. *J. Perinatol.* 40 909–915. 10.1038/s41372-020-0632-2 32086439 PMC7224016

[B13] Ferrara L. Bidiwala A. Sher I. Pirzada M. Barlev D. Islam S. (2017). Effect of nasal continuous positive airway pressure on the pharyngeal swallow in neonates. *J. Perinatol.* 37 398–403. 10.1038/jp.2016.229 28055023

[B14] Gewolb I. H. Vice F. L. (2006). Maturational changes in the rhythms, patterning, and coordination of respiration and swallow during feeding in preterm and term infants. *Dev. Med. Child Neurol.* 48 589–594. 10.1017/S001216220600123X 16780629

[B15] Gewolb I. H. Vice F. L. Schweitzer-Kenney E. L. Taciak V. L. Bosma J. F. (2001). Developmental patterns of rhythmic suck and swallow in preterm infants. *Dev. Med. Child. Neurol.* 43 22–27. 10.1017/s0012162201000044 11201418

[B16] Glackin S. J. O’Sullivan A. George S. Semberova J. Miletin J. (2017). High flow nasal cannula versus NCPAP, duration to full oral feeds in preterm infants: a randomised controlled trial. *Arch. Dis. Child. Fetal Neonatal* 102 329–332. 10.1136/archdischild-2016-311388 28011792

[B17] Hanin M. Nuthakki S. Malkar M. B. Jadcherla S. R. (2015). Safety and efficacy of oral feeding in infants with BPD on nasal CPAP. *Dysphagia* 30 121–127. 10.1007/s00455-014-9586-x 25380678 PMC4800480

[B18] Hasan S. U. Lodha A. K. Yusuf K. Dalgleish S. (2020). Physiological basis of neonatal aerodigestive difficulties in chronic lung disease. *Clin. Perinatol.* 47 277–299. 10.1016/j.clp.2020.03.001 32439112

[B19] Hatch L. D. Scott T. A. Walsh W. F. Goldin A. B. Blakely M. L. Patrick S. W. (2018). National and regional trends in gastrostomy in very low birth weight infants in the USA: 2000-2012. *J. Perinatol.* 38 1270–1276. 10.1038/s41372-018-0145-4 29925865 PMC6195828

[B20] Hiorns M. P. Ryan M. M. (2006). Current practice in paediatric videofluoroscopy. *Pediatr. Radiol.* 36 911–919. 10.1007/s00247-006-0124-3 16552584

[B21] Ianacone D. C. Gnadt B. J. Isaacson G. (2016). Ex vivo ovine model for head and neck surgical simulation. *Am. J. Otolaryngol.* 37 272–278. 10.1016/j.amjoto.2016.01.015 27178523

[B22] Isaacson G. Ianacone D. C. Soliman A. M. (2016). Ex vivo ovine model for suspension microlaryngoscopy training. *J. Laryngol. Otol.* 130 939–942. 10.1017/S0022215116008756 27572497

[B23] Isaacson G. Ianacone D. C. Wolfson M. R. (2015). Ex vivo ovine model for pediatric flexible endoscopy training. *Int. J. Pediatr. Otorhinolaryngol.* 79 2196–2199. 10.1016/j.ijporl.2015.10.002 26514929

[B24] Jadcherla S. R. Hasenstab K. A. Sitaram S. Clouse B. J. Slaughter J. L. Shaker R. (2016). Effect of nasal noninvasive respiratory support methods on pharyngeal provocation-induced aerodigestive reflexes in infants. *Am. J. Physiol.* 310 1006–1014. 10.1152/ajpgi.00307.2015 27012774 PMC4935482

[B25] Jadcherla S. R. Khot T. Moore R. Malkar M. Gulati I. K. Slaughter J. L. (2017). Feeding methods at discharge predict longterm feeding and neurodevelopmental outcomes in preterm infants referred for gastrostomy evaluation. *J. Pediatr.* 181 125–130.e1. 10.1016/j.jpeds.2016.10.065 27939123 PMC5724518

[B26] Lainwala S. Kosyakova N. Power K. Hussain N. Moore J. E. Hagadorn J. I. (2020). Delayed achievement of oral feedings is associated with adverse neurodevelopmental outcomes at 18 to 26 months follow-up in preterm infants. *Am. J. Perinatol.* 37 483–490. 10.1055/s-0039-1681059 30822799

[B27] Lau C. Smith E. O. Schanler R. J. (2003). Coordination of suck-swallow and swallow respiration in preterm infants. *Acta Pædiatr.* 92 721–727. 12856985

[B28] Layly J. Marmouset F. Chassagnon G. Bertrand P. Sirinelli D. Cottier J. P. (2020). Can we reduce frame rate to 15 images per second in pediatric videofluoroscopic swallow studies? *Dysphagia* 35 296–300. 10.1007/s00455-019-10027-8 31165922

[B29] Lemyre B. Davis P. G. De Paoli A. G. Kirpalani H. (2017). Nasal intermittent positive pressure ventilation (NIPPV) versus nasal continuous positive airway pressure (NCPAP) for preterm neonates after extubation. *Cochrane Database Syst. Rev.* 2:CD003212. 10.1002/14651858.CD003212.pub3 PMC6464652 28146296

[B30] Lemyre B. Laughon M. Bose C. Davis P. G. (2016). Early nasal intermittent positive pressure ventilation (NIPPV) versus early nasal continuous positive airway pressure (NCPAP) for preterm infants. *Cochrane Database Syst. Rev.* 12:CD005384. 10.1002/14651858.CD005384.pub2 27976361 PMC6463790

[B31] Maastrup R. Bojesen S. N. Kronborg H. Hallström I. (2012). Breastfeeding support in neonatal intensive care: a national survey. *J. Hum. Lact.* 28 370–379. 10.1177/0890334412440846 22674965

[B32] Mahajan V. Tiwari M. Arya A. Tiwari A. Chawla D. Saini S. S. (2016). Clinical predictors of hospital admission in acute lower respiratory tract infection in 2 months to 2-year-old children. *Respirology* 21 350–356. 10.1111/resp.12684 26611176

[B33] Mayerl C. J. Gould F. D. H. Bond L. E. Stricklen B. M. Buddington R. K. German R. Z. (2019). Preterm birth disrupts the development of feeding and breathing coordination. *J. Appl. Physiol.* 126 1681–1686. 10.1152/japplphysiol.00101.2019 31018743 PMC6620663

[B34] Medhurst A. (2005). Feeding protocols to improve the transition from gavage feeding to oral feeding in healthy premature infants: a systematic review. *Health Care Rep.* 3:1.

[B35] Mizuno K. Ueda A. (2003). The maturation and coordination of sucking, swallowing, and respiration in preterm infants. *J. Pediatr.* 142 36–40. 10.1067/mpd.2003.mpd0312 12520252

[B36] Nasef N. El-Gouhary E. Schurr P. Reilly M. Beck J. Dunn M. (2015). High-flow nasal cannulae are associated with increased diaphragm activation compared with nasal continuous positive airway pressure in preterm infants. *Acta Paediatr.* 104 337–343. 10.1111/apa.12998 25759095

[B37] Nyqvist K. H. (2008). Early attainment of breastfeeding competence in very preterm infants. *Acta Paediatr.* 97 776–781. 10.1111/j.1651-2227.2008.00810.x 18460108

[B38] Park J. Knafl G. Thoyre S. Brandon D. (2015). Factors associated with feeding progression in extremely preterm infants. *Nurs. Res.* 64 159–167. 10.1097/NNR.0000000000000093 25932696 PMC4418036

[B39] Pickens D. L. Schefft G. Thach B. T. (1988). Prolonged apnea associated with upper airway protective reflexes in apnea of prematurity. *Am. Rev. Respir. Dis.* 137 113–118. 10.1164/ajrccm/137.1.113 3337450

[B40] Samson N. Dumont S. Specq M. L. Praud J. P. (2011). Radio telemetry devices to monitor breathing in non-sedated animals. *Respir. Physiol. Neurobiol.* 179 111–118. 10.1016/j.resp.2011.09.008 21964163

[B41] Samson N. Michaud A. Othman R. Nadeau C. Nault S. Cantin D. (2017). Nasal continuous positive airway pressure influences bottle-feeding in preterm lambs. *Pediatr. Res.* 82 926–933. 10.1038/pr.2017.162 28700565

[B42] Samson N. Nadeau C. Cantin D. Farkouh R. Robinson M. Elnazir P. (2021). Respiratory activity of the cricopharyngeus muscle in the neonatal period. *Respir. Physiol. Neurobiol.* 290 103671. 10.1016/j.resp.2021.103671 33813048

[B43] Samson N. Nadeau C. Vincent L. Cantin D. Praud J. P. (2018). Effects of nasal continuous positive airway pressure and high-flow nasal cannula on sucking, swallowing, and breathing during bottle-feeding in lambs. *Front. Pediatr.* 5:296. 10.3389/fped.2017.00296 29387680 PMC5776098

[B44] Shimizu D. Araki S. Kawamura M. Kuwamura M. Suga S. Miyake F. (2019). Impact of high flow nasal cannula therapy on oral feeding in very low birth weight infants with chronic lung disease. *J. UOEH* 41 131–138. 10.7888/juoeh.41.131 31292356

[B45] St-Hilaire M. Samson N. Nsegbe E. Duvareille C. Moreau-Bussière F. Micheau P. (2007). Postnatal maturation of laryngeal chemoreflexes in the preterm lamb. *J. Appl. Physiol.* 102 1429–1438. 10.1152/japplphysiol.00977.2006 17170207

[B46] Thach B. T. (2008). Some aspects of clinical relevance in the maturation of respiratory control in infants. *J. Appl. Physiol.* 104 1828–1834. 10.1152/japplphysiol.01288.2007 18420716

[B47] Walsh M. C. Bell E. F. Kandefer S. Saha S. Carlo W. A. D’angio C. T. (2017). Neonatal outcomes of moderately preterm infants compared to extremely preterm infants. *Pediatr. Res.* 82 297–304. 10.1038/pr.2017.46 28419085 PMC5552426

