# Corrigendum: Safety of Bottle-Feeding Under Nasal Respiratory Support in Preterm Lambs With and Without Tachypnoea

**DOI:** 10.3389/fphys.2022.854173

**Published:** 2022-02-22

**Authors:** Basma Fathi Elsedawi, Nathalie Samson, Charlène Nadeau, Kristien Vanhaverbeke, Nam Nguyen, Charles Alain, Etienne Fortin-Pellerin, Jean-Paul Praud

**Affiliations:** ^1^Neonatal Respiratory Research Unit, Department of Pediatrics, Department of Physiology, University of Sherbrooke, Sherbrooke, QC, Canada; ^2^Department of Human Anatomy and Embryology, Faculty of Medicine, Zagazig University, Zagazig, Egypt; ^3^Laboratory of Experimental Medicine and Pediatrics, Infla-Med Centre of Excellence, University of Antwerp, Antwerp, Belgium; ^4^Department of Pediatrics, Antwerp University Hospital, Edegem, Belgium; ^5^Faculty of Human Medicine, Paracelsus Medical University, Nuremberg, Germany

**Keywords:** lamb, non-invasive respiratory support, oral feeding, preterm, sucking–swallowing–breathing coordination

An author name was incorrectly spelled as Basma Fathi Elsewadi in the authors list and in the financial support. The correct spelling is Basma Fathi Elsedawi.

The authors apologize for this error and state that this does not change the scientific conclusions of the article in any way. The original article has been updated.

## Publisher's Note

All claims expressed in this article are solely those of the authors and do not necessarily represent those of their affiliated organizations, or those of the publisher, the editors and the reviewers. Any product that may be evaluated in this article, or claim that may be made by its manufacturer, is not guaranteed or endorsed by the publisher.

