# Supplementary material for: Safety of Bottle-Feeding Under Nasal Respiratory Support in Preterm Lambs With and Without Tachypnoea
Source: Front Physiol. 2022 Jan 3;12:785086. doi: 10.3389/fphys.2021.785086 (PMC8762202; doi:10.3389/fphys.2021.785086)
Supplement: Supplementary file 1 [file Data_Sheet_1.docx]

**SUPPLEMENTAL DATA**

**Table S1: The percentage of bottles which had to be offered more than once for the lamb to drink its whole content, and the percentage of bottles in which the preterm lamb was held by an experimenter, are presented for all experimental conditions on days 7-8 and days 13-14**

|  | **CTRL (%)** | **nCPAP (%)** | **HFNC (%)** | **HFNCcpap (%)** |
| --- | --- | --- | --- | --- |
| ***PERCENTAGE OF BOTTLES OFFERED MORE THAN ONCE*** | | | | |
| **On days 7-8 without tachypnoea** | 44 | 40 | 53 | 43 |
| **On days 7-8 with tachypnoea** | 52 | 38 | 52 | 53 |
| **On days 13-14 without tachypnoea** | 56 | 49 | 54 | 54 |
| **On days 13-14 with tachypnoea** | 55 | 53 | 56 | 48 |
| ***PERCENTAGE OF BOTTLES HELD BY AN EXPERIMENTER*** | | | | |
| **On days 7-8 without tachypnoea** | 50 | 67 | 29 | 36 |
| **On days 7-8 with tachypnoea** | 20 | 64 | 36 | 23 |
| **On days 13-14 without tachypnoea** | 25 | 71 | 18 | 36 |
| **On days 13-14 with tachypnoea** | 25 | 56 | 45 | 36 |

Absolute number of lambs with successful recordings on postnatal days 7-8: control (n = 15), nCPAP (n = 14 with and n = 15 without tachypnoea), HFNC (n = 13 with and n = 14 without tachypnoea), and HFNCcpap (n = 14 with and n = 15 without tachypnoea). On postnatal days 13-14: control (n = 12), nCPAP (n = 9 with and n = 11 without tachypnoea), HFNC (n = 11), and HFNCcpap (n = 11).

**Table S2: Safety, efficiency and coordination of bottle feeding in preterm lambs without tachypnoea on postnatal days 7-8**

|  | **CTRL** | **nCPAP** | **HFNC** | **HFNCcpap** |
| --- | --- | --- | --- | --- |
|  | **Median (Q1, Q3)** | **Median (Q1, Q3)** | **Median (Q1, Q3)** | **Median (Q1, Q3)** |
|  | **n = 15** | **n = 15** | **n = 14** | **n = 15** |
| ***SAFETY OF BOTTLE FEEDING*** | | | | |
| **Heart slowing (no.)** | 0 (0,0) | 0 (0,0) | 0 (0,0) | 0 (0,0) |
| **Bradycardia (no.)** | 0 (0,0) | 0 (0,0) | 0 (0,0) | 0 (0,0) |
| **Minimal heart rate (bpm)** | 169 (134,179) | 183 (168,196) *#+ | 176 (168,185) | 178 (155,200) |
| **Heart rate** **decrease (%)** | 21 (15,30) | 21 (14,23) | 19 (13,21) * | 22 (16,27) |
| **Inhibition time (s)** | 0 (0,0) | 0 (0,0) | 0 (0,0) | 0 (0,0) |
| **Minimal SpO_2_ (%)** | 94 (90,96) | 94 (93,98) | 93 (91,96) | 95 (92,97) |
| **SpO_2_ decrease (%)** | 1 (0,5) | 1 (1, 3) | 2 (1,5) | 3 (1,5) |
| **Cough (no.)** | 0 (0,0) | 0 (0, 0) | 0 (0,0) | 0 (0, 0) |
| **Laryngeal penetration (no.)** | 0 (0,0) | 0 (0,0) | 0 (0,0) | 0 (0,0) |
| **Tracheal aspiration (no.)** | 0 (0,0) | 0 (0,0) | 0 (0,0) | 0 (0,0) |
| ***EFFICIENCY OF BOTTLE FEEDING*** | | | | |
| **Rate of milk transfer** **(mL**•**s^-1^)** | 0.6 (0.5,1.0) | 0.5 (0.4,0.7) | 0.5 (0.4,0.7) | 0.5 (0.4,0.7) |
| **Sucks**•**mL^-1^** | 3 (3,4) | 3 (3,5) | 4 (3,5) | 3 (3,5) |
| **Swallows**•**mL^-1^** | 2 (2,3) | 3 (2,3) | 3 (2,4) | 3 (2,3) |
| **Sucking amplitude (mmHg)** | 56 (32,87) | 48 (37,66) | 63 (50,74) | 48 (37,66) |
| ***SU-SW-BR COORDINATION*** | | | | |
| **SW in apnoea (%)** | 25 (0,45) | 2 (0,22) | 15 (0, 38) | 22 (0, 22) |
| **Feeding time in apnoea (%)** | 15 (0,32) | 1 (0,12) | 7 (0, 28) | 11 (0,12) |
| **SU-SU interval (s)** | 0.40 (0.34,0.54) | 0.47 (0.39,0.59) | 0.36 (0.33,0.60) | 0.45 (0.38,0.53) |
| **SU-SW interval (s)** | 0.30 (0.24,0.34) | 0.31 (0.28,0.35) | 0.28 (0.26,0.32) | 0.31 (0.28,0.34) |
| **SW-SW interval (s)** | 0.56 (0.43,0.73) | 0.68 (0.54,0.95) | 0.58 (0.41,0.74) | 0.63 (0.49,0.76) |
| **SW-BR interval (s)** | 1.16 (0.75,1.77) | 0.88 (0.74,1.04) | 0.96 (0.78,2.00) | 1.06 (0.90,1.58) |
| **SU-SU COV** | 0.52 (0.30,0.76) | 0.76 (0.61,0.93) | 0.51 (0.33,0.72) | 0.62 (0.46,0.86) |
| **SU-SW COV** | 0.25 (0.15,0.53) | 0.39 (0.18,0.53) | 0.25 (0.15,0.33) | 0.32 (0.19,0.39) |
| **SW-SW COV** | 0.51 (0.41,0.62) | 0.60 (0.48,0.76) | 0.56 (0.46,0.83) | 0.62 (0.52,0.80) |
| **SW-BR COV** | 0.66 (0.53,0.80) | 0.64 (0.57,0.76) | 0.73 (0.58,0.89) | 0.78 (0.59,0.92) * |

CTRL = control condition (no nasal respiratory support); nCPAP = nasal continuous positive airway pressure; HFNC = high-flow nasal cannula; HFNCcpap = high-flow nasal cannula with an end-expiratory tracheal pressure of 6 cmH_2_O; (Q1, Q3) = first and third quartiles; HR = heart rate; RR = respiratory rate; SpO_2_ = oxygen saturation; * p < 0.05 vs. control; # p < 0.05 vs. HFNC; + p < 0.05 vs HFNCcpap.

|  | **CTRL** | **nCPAP** | **HFNC** | **HFNCcpap** |
| --- | --- | --- | --- | --- |
|  | **Median (Q1, Q3)** | **Median (Q1, Q3)** | **Median (Q1, Q3)** | **Median (Q1, Q3)** |
|  | **n = 14** | **n = 14** | **n = 12** | **n = 12** |
| ***BASELINE*** | | | | |
| **Tracheal pressure (cmH_2_O)** | 0.6 (0.2,0.9) ^#+ | 6.2 (6.1,6.3) | 2.5 (1.9,3.0) ^+ | 6.0 (5.8,6.1) |
| **HR (bpm)** | 204 (188,221) | 220 (200,231) # | 194 (182,201) | 207 (194,226) |
| **RR (min^-1^)** | 54 (48,66) | 54 (44,59) | 48 (42,54) * | 48 (42,54) |
| **SpO_2_ (%)** | 96 (95,97) | 97 (95,99) | 98 (96,98) | 97 (95,98) |
| **PaO_2_ (mmHg)** | 88 (85,94) | 93 (85,95) | 89 (86,92) | 94 (84,102) |
| **PaCO_2_ (mmHg)** | 34 (31,36) | 34 (32,36) | 32 (28,35) | 34 (31,36) |
| **pH** | 7.41 (7.35,7.42) | 7.40 (7.37,7.44) | 7.41 (7.39,7.43) | 7.41 (7.38,7.45) |
| ***1 MIN AFTER FEEDING*** | | | | |
| **HR (bpm)** | 217 (202,230) | 222 (211,229) | 216 (202,225) | 230 (201,237) |
| **RR (min^-1^)** | 63 (51,75) ^#+ | 45 (38,48) | 45 (41,60) | 48 (41,56) |
| **SpO_2_ (%)** | 96 (95,96) | 97 (93,99) | 96 (94,97) | 96 (94,97) |
| **PaO_2_ (mmHg)** | 98 (89,104) | 103 (95,106) | 96 (90,101) | 98 (91,105) |
| **PaCO_2_ (mmHg)** | 32 (30,33) | 34 (31,36) | 32 (30,36) | 33 (32,35) |
| **pH** | 7.40 (7.38,7.45) | 7.38 (7.37,7.43) | 7.42 (7.37,7.43) | 7.39 (7.37,7.43) |

**Table S3: Arterial blood gases in preterm lambs without tachypnoea on postnatal days 7-8**

PaO_2_ = arterial oxygen pressure; PaCO_2_ = arterial CO_2_ pressure; see table 1 for other abbreviations. * p < 0.05 vs. control; ^ p < 0.05 vs. nCPAP; # p < 0.05 vs. HFNC; + p < 0.05 vs HFNCcpap.

**Table S4: Safety, efficiency, and coordination of bottle feeding in preterm lambs without tachypnoea on postnatal days 13-14**

|  | **CTRL** | **nCPAP** | **HFNC** | **HFNCcpap** |
| --- | --- | --- | --- | --- |
|  | **Median (Q1, Q3)** | **Median (Q1, Q3)** | **Median (Q1, Q3)** | **Median (Q1, Q3)** |
|  | **n = 12** | **n = 11** | **n = 11** | **n = 11** |
|  |  |  |  |  |
| ***SAFETY OF BOTTLE FEEDING*** | | | | |
| **Heart slowing (no.)** | 0 (0,0) | 0 (0,0) | 0 (0,0) | 0 (0,0) |
| **Bradycardia (no.)** | 0 (0,0) | 0 (0,0) | 0 (0,0) | 0 (0,0) |
| **Minimal heart rate (bpm)** | 163 (144,180) | 181 (175,198) | 153 (108,182) ^+ | 171 (150,195) |
| **Heart rate** **decrease (%)** | 17 (10,26) | 17 (10,28) | 17 (13,40) | 13 (11,20) |
| **Inhibition time (s)** | 0 (0,1) | 0 (0,0) # | 0 (0,1) | 0 (0,0) |
| **Minimal SpO_2_ (%)** | 95 (92,97) | 94 (93,96) | 95 (93,96) | 95 (86,97) |
| **SpO_2_ decrease (%)** | 4 (2,7) | 2 (1, 4) | 2 (1,3) | 2 (0,8) |
| **Cough (no.)** | 0 (0,0) | 0 (0,0) | 0 (0,0) | 0 (0,0) |
| **Laryngeal penetration (no.)** | 0 (0,0) | 0 (0,0) | 0 (0,0) | 0 (0,0) |
| **Tracheal aspiration (no.)** | 0 (0,0) | 0 (0,0) | 0 (0,0) | 0 (0,0) |
| ***EFFICIENCY OF BOTTLE FEEDING*** | | | | |
| **Rate of milk transfer** **(mL**•**s^-1^)** | 0.5 (0.4,1.0) | 0.7 (0.4,1.0) | 0.6 (0.4,0.9) | 0.5 (0.5,0.9) |
| **Sucks**•**mL^-1^** | 3 (2,4) | 3 (2,4) | 4 (3,4) | 3 (2,5) |
| **Swallows**•**mL^-1^** | 2 (2,3) | 2 (1,3) | 2 (2,3) | 2 (2,3) |
| **Sucking amplitude (mmHg)** | 81 (62,106) | 82 (63,114) #+ | 91 (30,122) | 62 (46,81) |
| ***SU-SW-BR COORDINATION*** | | | | |
| **SW in apnoea (%)** | 21 (0,36) | 14 (0,35) | 0 (0,30) | 20 (0,54) |
| **Feeding time in apnoea (%)** | 11 (0,23) | 6 (0,19) | 0 (0,20) | 12 (0, 42) |
| **SU-SU interval (s)** | 0.38 (0.34,0.47) | 0.51 (0.32,0.65) | 0.49 (0.37,0.79) | 0.45 (0.36,0.56) |
| **SU-SW interval (s)** | 0.28 (0.25,0.32) | 0.33 (0.28,0.40) | 0.32 (0.26,0.38) | 0.30 (0.25,0.36) |
| **SW-SW interval (s)** | 0.63 (0.48,0.85) | 0.86 (0.68,1.15) *#+ | 0.84 (0.54,1.04) * | 0.68 (0.46,0.96) *# |
| **SW-BR interval (s)** | 1.11 (0.88,1.44) | 0.99 (0.86,1.29) + | 0.98 (0.80,1.56) | 1.20 (0.79,1.75) |
| **SU-SU COV** | 0.55 (0.24,0.78) | 0.49 (0.32,1.02) *+ | 0.71 (0.42,0.96) | 0.54 (0.39,0.80) |
| **SU-SW COV** | 0.31 (0.21,0.43) | 0.37 (0.23,0.61) | 0.38 (0.30,0.50) | 0.33 (0.18,0.53) |
| **SW-SW COV** | 0.47 (0.38,0.68) | 0.71 (0.48,0.89) | 0.51 (0.39,0.68) | 0.62 (0.48,0.71) |
| **SW-BR COV** | 0.68 (0.56,0.77) | 0.65 (0.56,0.80) | 0.58 (0.50,0.74) | 0.64 (0.56,0.73) |

For abbreviations see table 1. * p < 0.05 vs. control; ^ p < 0.05 vs. nCPAP; # p < 0.05 vs. HFNC; + p < 0.05 vs HFNCcpap.

**Table S5: Arterial blood gases in preterm lambs without tachypnoea on postnatal days 13-14**

|  | **CTRL** | **nCPAP** | **HFNC** | **HFNCcpap** |
| --- | --- | --- | --- | --- |
|  | **Median (Q1, Q3)** | **Median (Q1, Q3)** | **Median (Q1, Q3)** | **Median (Q1, Q3)** |
|  | **n = 11** | **n = 9** | **n = 10** | **n = 10** |
| ***BASELINE*** | | | | |
| **Tracheal pressure (cmH_2_O)** | 0.7 (0.3,0.8) ^#+ | 6.1 (5.8,6.3) | 2.5 (2.3,2.8) ^+ | 6 (5.8,6.0) |
| **HR (bpm)** | 195 (176,229) | 205 (179,228) | 191 (181,200) | 193 (176,214) |
| **RR (min^-1^)** | 60 (48,72) #+ | 48 (42,53) # | 33 (26,47) | 36 (36,47) |
| **SpO_2_ (%)** | 97 (96,98) | 98 (96,98) | 98 (97,99) | 96 (96,99) |
| **PaO_2_ (mmHg)** | 86 (83,92) ^#+ | 99 (94,101) | 101 (94,102) | 94 (90,101) |
| **PaCO_2_ (mmHg)** | 35 (34,37) # | 36 (34,38) # | 33 (32,34) | 33 (31,37) |
| **pH** | 7.44 (7.42,7.45) | 7.42 (7.41,7.43) | 7.45 (7.41,7.46) | 7.43 (7.37,7.44) |
| ***1 MIN AFTER FEEDING*** | | | | |
| **HR (bpm)** | 195 (180,217) | 216 (193,233) | 198 (177,216) | 205 (190,232) |
| **RR (min^-1^)** | 60 (48,66) ^#+ | 36 (32,42) | 30 (30,42) | 42 (36,42) |
| **SpO_2_ (%)** | 97 (96,98) | 97 (96,99) | 97 (95,98) | 97 (95,98) |
| **PaO_2_ (mmHg)** | 100 (88,108) | 101 (97,106) | 99 (94,102) | 103 (100,107) |
| **PaCO_2_ (mmHg)** | 31 (29,34) #^ | 34 (30,36) + | 32 (31,37) | 30 (29,32) # |
| **pH** | 7.46 (7.44,7.48) | 7.44 (7.43,7.45) | 7.43 (7.42,7.45) | 7.44 (7.41,7.46) |

For abbreviations see table 1. ^ p < 0.05 vs. nCPAP; # p < 0.05 vs. HFNC; + p < 0.05 vs HFNCcpap.

**Table S6: Respiratory rate observed in lambs on postnatal days 7-8 during the experiments with induced tachypnoea**

|  | **CTRL** | **nCPAP6** | **HFNC** | **HFNCcpap** |
| --- | --- | --- | --- | --- |
|  | **Median (Q1, Q3)** | **Median (Q1, Q3)** | **Median (Q1, Q3)** | **Median (Q1, Q3)** |
|  | **n = 15** | **n = 14** | **n = 13** | **n = 14** |
| **Baseline** | 54 (44, 65) | 54 (42, 54) | 54 (42, 63) | 54 (48, 60) |
| **Standardized tachypnoea** | 75 (66, 84) ^¥^ | 72 (63, 83) ^¥, 🟁^ | 78 (62, 86) ^¥^ | 78 (72, 87) ^¥, 🟁^ |
| **With nasal respiratory support** | - | 54 (48, 63) | 66 (51, 72) ^¥^ | 60 (51, 70) ^¥^ |

**Baseline:** before imposition of tachypnoea; **Standardized tachypnoea:** after imposition of tachypnoea, but before application of nasal respiratory support; **With nasal respiratory support:** after imposition of tachypnoea and application of nasal respiratory support.

**CTRL**, control condition without nasal respiratory support; **nCPAP**, nasal continuous positive airway pressure at 6 cmH_2_O; **HFNC**, high-flow nasal cannulae at 7 L**•**min^-1^; **HFNCcpap**, high-flow nasal cannulae at 7 L**•**min^-1^ with end-expiratory tracheal pressure at 6 cmH_2_O. ^¥^ p < 0.05 vs. baseline; 🟁p < 0.05 vs. with nasal respiratory support.

**Table S7: Safety, efficiency and coordination of bottle feeding in preterm lambs with tachypnoea on postnatal days 7-8**

|  | **CTRL** | **nCPAP** | **HFNC** | **HFNCcpap** |
| --- | --- | --- | --- | --- |
|  | **Median (Q1, Q3)** | **Median (Q1, Q3)** | **Median (Q1, Q3)** | **Median (Q1, Q3)** |
|  | **n = 15** | **n = 14** | **n = 13** | **n = 14** |
| ***SAFETY OF BOTTLE FEEDING*** | | | | |
| **Heart slowing (no.)** | 0 (0,0) | 0 (0,0) | 0 (0,0) | 0 (0,0) |
| **Bradycardia (no.)** | 0 (0,0) | 0 (0,0) | 0 (0,0) | 0 (0,0) |
| **Minimal heart rate (bpm)** | 168 (145,185) | 186 (178,196) *#+ | 166 (151,169) | 175 (159,191) * |
| **Heart rate decrease (%)** | 20 (10,26) | 20 (12,23) # | 23 (18,27) | 21 (16,24) |
| **Inhibition time (s)** | 0 (0,0) | 0 (0,0) | 0 (0,0) | 0 (0,0) |
| **Minimal SpO_2_ (%)** | 91 (85,4) | 93 (90,94) + | 90 (85,93) | 89 (82,93) |
| **SpO_2_ decrease (%)** | 4 (2,9) | 2 (1,5) *+ | 2 (1,8) + | 6 (3,13) |
| **Cough (no.)** | 0 (0,0) | 0 (0,0) | 0 (0,0) | 0 (0,0) |
| **Laryngeal penetration (no.)** | 0 (0,0) | 0 (0,0) | 0 (0,0) | 0 (0,0) |
| **Tracheal aspiration (no.)** | 0 (0,0) | 0 (0,0) | 0 (0,0) | 0 (0,0) |
| ***EFFICIENCY OF BOTTLE FEEDING*** | | | | |
| **Rate of milk transfer (mL•s^-1^)** | 0.5 (0.4,0.7) | 0.5 (0.4,0.7) | 0.6 (0.4,0.7) | 0.5 (0.4,0.9) |
| **Sucks•mL^-1^** | 4 (3,5) | 4 (3,4) | 3 (2,4) | 4 (2,4) |
| **Swallows•mL^-1^** | 3 (2,4) | 3 (2,4) | 3 (2,4) | 2 (2,3) |
| **Sucking amplitude (mmHg)** | 61 (37,86) | 64 (53,70) + | 59 (41,66) | 49 (33,65) |
| ***SU-SW-BR COORDINATION*** | | | | |
| **SW in apnoea (%)** | 11 (0,28) | 8 (0,29) | 15 (0,33) | 0 (0,32) |
| **Feeding time in apnoea (%)** | 6 (0,19) | 3 (0,14) | 9 (0,18) | 0 (0,20) |
| **SU-SU interval (s)** | 0.44 (0.33,0.59) | 0.54 (0.37,0.61) # | 0.42 (0.36,0.51) | 0.51 (0.36,0.69) |
| **SU-SW interval (s)** | 0.31 (0.26,0.33) | 0.31 (0.29,0.37) | 0.29 (0.27,0.33) | 0.35 (0.26,0.39) |
| **SW-SW interval (s)** | 0.68 (0.47,0.96) | 0.72 (0.48,1.00) | 0.57 (0.44,0.90) | 0.80 (0.50,1.25) |
| **SW-BR interval (s)** | 0.84 (0.64,1.31) | 0.85 (0.68,1.01) | 0.88 (0.76,1.45) | 0.74 (0.66,1.40) |
| **SU-SU COV** | 0.51 (0.41,0.86) | 0.62 (0.47,0.89) | 0.44 (0.20,0.63) *^+ | 0.56 (0.34,0.90) |
| **SU-SW COV** | 0.24 (0.20,0.40) | 0.38 (0.21,0.44) | 0.23 (0.14,0.31) | 0.25 (0.14,0.44) |
| **SW-SW COV** | 0.67 (0.51,0.77) | 0.71 (0.49,0.94) | 0.45 (0.35,0.79) | 0.57 (0.40,0.80) |
| **SW-BR COV** | 0.68 (0.51,0.86) | 0.57 (0.47,0.78) | 0.70 (0.50,0.83) | 0.59 (0.52,0.73) |

For abbreviations see table 1. * p < 0.05 vs. control; ^ p < 0.05 vs. nCPAP; # p < 0.05 vs. HFNC; + p < 0.05 vs HFNCcpap.

|  | **CTRL** | **nCPAP** | **HFNC** | **HFNCcpap** |
| --- | --- | --- | --- | --- |
|  | **Median (Q1, Q3)** | **Median (Q1, Q3)** | **Median (Q1, Q3)** | **Median (Q1, Q3)** |
|  | **n = 11** | **n = 8** | **n = 9** | **n = 9** |
| ***BASELINE*** | | | | |
| **Tracheal pressure (cmH_2_O)** | 0.3 (0.2,0.8) ^#+ | 6.0 (5.9,6.3) | 2.2 (1.8,2.6) ^+ | 5.9 (5.8,6.1) |
| **HR (bpm)** | 201 (187,208) | 223 (192,229) | 205 (196,225) | 215 (200,228) |
| **RR (min^-1^)** | 66 (60,78) + | 60 (54,60) # | 66 (60,72) | 60 (54,60) |
| **SpO_2_ (%)** | 96 (95,98) | 96 (94,97) | 94 (93,95) | 96 (95,97) |
| **PaO_2_ (mmHg)** | 85 (82,92) #+ | 85 (73,92) # | 76 (71,77) | 76 (73,77) |
| **PaCO_2_ (mmHg)** | 35 (34,37) | 35 (34,37) | 32 (31,33) | 34 (32,37) |
| **pH** | 7.42 (7.38,7.43) | 7.40 (7.35,7.43) | 7.41 (7.37,7.43) | 7.41 (7.39,7.43) |
| ***1 MIN AFTER FEEDING*** | | | | |
| **HR (bpm)** | 214 (191,219) | 232 (219,235) * | 224 (205,231) | 231 (204,233) |
| **RR (min^-1^)** | 75 (62,87) ^+ | 60 (54,60) | 69 (60,72) | 54 (54,65) |
| **SpO_2_ (%)** | 97 (95,97) | 96 (94,97) | 95 (93,97) | 95 (89,97) |
| **PaO_2_ (mmHg)** | 83 (80,87) | 81 (78,87) | 77 (73,80) | 75 (72,78) |
| **PaCO_2_ (mmHg)** | 35 (31,37) | 35 (33,39) | 34 (32,36) | 32 (30,36) |
| **pH** | 7.37 (7.35,7.40) | 7.43 (7.35,7.44) | 7.41 (7.36,7.43) | 7.42 (7.35,7.42) |

**Table S8: Arterial blood gases in preterm lambs with tachypnoea on postnatal days 7-8**

For abbreviations see table 1. * p < 0.05 vs. control; ^ p < 0.05 vs. nCPAP; # p < 0.05 vs. HFNC; + p < 0.05 vs HFNCcpap.

**Table S9: Safety, efficiency, and coordination of bottle feeding in preterm lambs with tachypnoea on postnatal days 13-14**

|  | **Control** | **nCPAP** | **HFNC** | **HFNCcpap** |
| --- | --- | --- | --- | --- |
|  | **Median (Q1, Q3)** | **Median (Q1, Q3)** | **Median (Q1, Q3)** | **Median (Q1, Q3)** |
|  | **n = 12** | **n = 9** | **n = 11** | **n = 11** |
| ***SAFETY OF BOTTLE FEEDING*** | | | | |
| **Heart slowing (no.)** | 0 (0,0) | 0 (0,0) | 0 (0,0) | 0 (0,0) |
| **Bradycardia (no.)** | 0 (0,0) | 0 (0,0) | 0 (0,0) | 0 (0,0) |
| **Minimal heart rate (bpm)** | 163 (153,172) | 170 (156,186) | 160 (151,182) | 159 (143,172) |
| **Heart rate** **decrease (%)** | 17 (10,21) | 20 (13,23) | 19 (15,24) | 19 (16,25) |
| **Inhibition time (s)** | 0 (0,0) | 0 (0,0) | 0 (0,0) | 0 (0,0) |
| **Minimal SpO_2_ (%)** | 90 (84,93) | 94 (91,95) *# | 89 (86,92) | 95 (86,96) * |
| **SpO_2_ decrease (%)** | 6 (3,10) | 2 (1,3) * | 5 (1,7) | 1 (0,6) |
| **Cough (no.)** | 0 (0,0) | 0 (0,0) | 0 (0,0) | 0 (0,0) |
| **Laryngeal penetration (no.)** | 0 (0,0) | 0 (0,0) | 0 (0,0) | 0 (0,0) |
| **Tracheal aspiration (no.)** | 0 (0,0) | 0 (0,0) | 0 (0,0) | 0 (0,0) |
| ***EFFICIENCY OF BOTTLE FEEDING*** | | | | |
| **Rate of milk transfer** **(mL•s^-1^)** | 0.7 (0.4,1.2) | 0.4 (0.4,0.7) | 0.6 (0.5,1.1) | 0.6 (0.6,1.1) |
| **Sucks•mL^-1^** | 3 (2,3) | 3 (2,3) | 3 (2,3) | 3 (2,4) |
| **Swallows•mL^-1^** | 2 (2,2) | 3 (1,3) | 2 (2,2) | 2 (2,2) |
| **Sucking amplitude (mmHg)** | 88 (51,107) | 54 (27,74) | 71 (57,93) | 68 (32,95) |
| ***SU-SW-BR COORDINATION*** | | | | |
| **SW in apnoea (%)** | 0 (0,37) | 9 (0,27) | 0 (0,6) | 0 (0,29) |
| **Feeding time in apnoea (%)** | 0 (0,27) | 5.5 (0,20) | 0 (0,4) | 0 (0,22) |
| **SU-SU interval (s)** | 0.46 (0.36,0.61) | 0.62 (0.35, 0.90) *# | 0.50 (0.42,0.59) | 0.50 (0.40,0.66) |
| **SU-SW interval (s)** | 0.31 (0.27,0.34) | 0.35 (0.26,0.42) | 0.30 (0.27,0.32) | 0.36 (0.29,0.44) *# |
| **SW-SW interval (s)** | 0.70 (0.51,1.01) | 0.92 (0.49,1.17) | 0.77 (0.57,0.99) | 0.88 (0.43,1.06) |
| **SW-BR interval (s)** | 0.86 (0.68,1.27) | 1.08 (0.89,1.58) * | 0.95 (0.86,1.22) | 0.90 (0.80,1.21) |
| **SU-SU COV** | 0.65 (0.45,0.76) | 0.74 (0.59,0.86) | 0.58 (0.33,0.92) | 0.50 (0.34,0.76) |
| **SU-SW COV** | 0.32 (0.22,0.40) | 0.36 (0.33,0.53) | 0.34 (0.18,0.45) | 0.39 (0.30,0.49) |
| **SW-SW COV** | 0.52 (0.42,0.66) | 0.53 (0.42,0.66) | 0.60 (0.38,0.76) | 0.46 (0.34,0.56) |
| **SW-BR COV** | 0.57 (0.51,0.78) | 0.60 (0.49,0.83) | 0.59 (0.52,0.80) | 0.57 (0.48,0.66) |

For abbreviations see table 1. * p < 0.05 vs. control; # p < 0.05 vs. HFNC.

|  | **CTRL** | **nCPAP** | **HFNC** | **HFNCcpap** |
| --- | --- | --- | --- | --- |
|  | **Median (Q1, Q3)** | **Median (Q1, Q3)** | **Median (Q1, Q3)** | **Median (Q1, Q3)** |
|  | **n = 11** | **n = 8** | **n = 10** | **n = 10** |
| ***BASELINE*** | | | | |
| **Tracheal pressure (cmH_2_O)** | 0.3 (0.1,0.5) ^#+ | 6 (6.0,6.2) | 3.2 (2.1,3.4) ^+ | 5.9 (5.8,6.1) |
| **HR (bpm)** | 191 (180,206) | 185.5 (178,191) | 186 (178,197) | 183.5 (167,211) |
| **RR (min^-1^)** | 66 (57,72) ^+# | 42 (41,46) # | 51 (48,66) | 42 (42, 3) |
| **SpO_2_ (%)** | 95 (94,97) | 96 (96,97) | 95 (94,96) | 97 (96,97) |
| **PaO_2_ (mmHg)** | 92 (86,96) #+ | 85 (83,88) # | 78 (73,80) | 81 (74,86) |
| **PaCO_2_ (mmHg)** | 35 (33,37) | 36 (34,38) | 35 (33,36) | 34 (33,36) |
| **pH** | 7.45 (7.43,7.48) | 7.41 (7.40,7.41) | 7.44 (7.43,7.45) | 7.43 (7.42,7.43) |
| ***1 MIN AFTER FEEDING*** | | | | |
| **HR (bpm)** | 194 (184,208) | 195 (183,199) | 193 (183,214) | 199 (187,217) |
| **RR (min^-1^)** | 72 (66,81) ^#+ | 48 (42,60) | 51 (44,59) | 48 (42,54) |
| **SpO_2_ (%)** | 94 (92,96) | 96 (95,97) | 95 (92,97) | 97 (96,99) |
| **PaO_2_ (mmHg)** | 82 (76,86) | 91 (82,98) | 81 (73,89) | 87 (79,88) |
| **PaCO_2_ (mmHg)** | 33 (32,34) | 35 (31,36) | 34 (31,35) | 32 (30,34) |
| **pH** | 7.44 (7.42,7.47) | 7.43 (7.42,7.46) | 7.44 (7.43,7.46) | 7.42 (7.41,7.44) |

**Table S10: Arterial blood gases in preterm lambs with tachypnoea on postnatal days 13-14**

For abbreviations see table 1. ^ p < 0.05 vs. nCPAP; # p < 0.05 vs. HFNC; + p < 0.05 vs. HFNCcpap.

**Table S11: Effect of tachypnoea on safety, efficiency, and coordination of bottle feeding on postnatal days 7-8 and 13-14**

|  | **P-values for tachypnoea** | |
| --- | --- | --- |
|  | **Postnatal days 7-8** | **Postnatal days 13-14** |
| ***SAFETY OF BOTTLE FEEDING*** | | |
| **Heart slowing (no.)** | 1 | 0.3 |
| **Bradycardia (no.)** | 0.3 | 0.3 |
| **Minimal heart rate (bpm)** | 0.7 | 0.7 |
| **Heart rate** **decrease (%)** | 0.5 | 0.2 |
| **Inhibition time (s)** | 0.3 | 0.9 |
| **Minimal SpO_2_ (%)** | **1.8e-11** | **2.8e-6** |
| **SpO_2_ decrease (%)** | **0.003** | 0.1 |
| **Cough (no.)** | 0.3 | 0.7 |
| ***EFFICIENCY OF BOTTLE FEEDING*** | | |
| **Rate of milk transfer** **(mL•s^-1^)** | 0.5 | 0.6 |
| **Sucks•mL^-1^** | 0.6 | 0.2 |
| **Swallows•mL^-1^** | 0.3 | 0.1 |
| **Sucking amplitude (mmHg)** | 0.8 | 0.4 |
| ***SU-SW-BR COORDINATION*** | | |
| **SW in apnoea (%)** | 0.2 | 0.1 |
| **Feeding time in apnoea (%)** | 0.3 | 0.2 |
| **SU-SU interval (s)** | 0.4 | 0.4 |
| **SU-SW interval (s)** | **0.003** | 0.5 |
| **SW-SW interval (s)** | **0.02** | 0.3 |
| **SW-BR interval (s)** | **0.006** | 0.06 |
| **SU-SU COV** | 0.3 | 0.5 |
| **SU-SW COV** | 0.4 | 0.4 |
| **SW-SW COV** | 0.7 | 0.1 |
| **SW-BR COV** | **0.04** | 0.6 |

For abbreviations see table 1. Bold results are significant differences (p < 0.05)

**Table S12: Effect of tachypnoea on arterial blood gases on postnatal days 7-8 and 13-14**

|  | **P-values for tachypnoea** | |
| --- | --- | --- |
|  | **Postnatal days 7-8** | **Postnatal days 13-14** |
| ***BASELINE*** | | |
| **Tracheal pressure (cmH_2_O)** | 0.08 | 0.9 |
| **HR (bpm)** | 0.7 | 0.09 |
| **RR (min^-1^)** | **9.4e-09** | **0.009** |
| **SpO_2_ (%)** | **0.01** | **0.008** |
| **PaO_2_ (mmHg)** | **2.9e-06** | **8.2e-9** |
| **PaCO_2_ (mmHg)** | 0.2 | 0.5 |
| **pH** | 1 | 0.3 |
| ***1 MIN AFTER FEEDING*** |  | |
| **HR (bpm)** | 0.3 | 0.1 |
| **RR (min^-1^)** | **7.8e-09** | **1.0e-08** |
| **SpO_2_ (%)** | 0.3 | **0.01** |
| **PaO_2_ (mmHg)** | **2.2e-16** | **2.2e-16** |
| **PaCO_2_ (mmHg)** | **0.04** | 0.05 |
| **pH** | **0.004** | 0.4 |

For abbreviations see table 1. Bold results are significant differences (p < 0.05).

**Table S13:** **End-expiratory tracheal pressure, with and without induced tachypnoea, during recordings on postnatal days 7-8 and 13-14**

|  | **CTRL** | **nCPAP6** | **HFNC** | **HFNCcpap** |
| --- | --- | --- | --- | --- |
|  | **Median (Q1, Q3)** | **Median (Q1, Q3)** | **Median (Q1, Q3)** | **Median (Q1, Q3)** |
| **At day 7-8 without tachypnoea** | 0.6 (0.2, 0.9) ^#+ | 6.2 (6.1, 6.3) | 2.5 (1.9, 3.0) ^+ | 6.0 (5.8, 6.1) |
| **At day 7-8 with tachypnoea** | 0.3 (0.2, 0.8) ^#+ | 6.0 (5.9, 6.3) | 2.2 (1.8, 2.6) ^+ | 5.9 (5.8, 6.1) |
| **At day 13-14 without tachypnoea** | 0.7 (0.3, 0.8) ^#+ | 6.1 (5.8, 6.3) | 2.5 (2.3, 2.8) ^+ | 6 (5.8, 6) |
| **At day 13-14 with tachypnoea** | 0.3 (0.1, 0.5) ^#+ | 6 (6, 6.2) | 3.2 (2.1, 3.4) ^+ | 5.9 (5.8, 6.1) |

**CTRL**, control condition without nasal respiratory support; **nCPAP**, nasal continuous positive airway pressure at 6 cmH_2_O; **HFNC**, high-flow nasal cannulae at 7 L•min^-1^; **HFNCcpap**, high-flow nasal cannulas at 7 L•min^-1^ with positive airway pressure at 6 cmH_2_O. ^ p < 0.05 vs. nCPAP; # p < 0.05 vs. HFNC; + p < 0.05 vs. HFNCcpap.

Number of lambs with successful recordings on postnatal days 7-8: control (n = 15), nCPAP (n = 14 with and n = 15 without tachypnoea), HFNC (n = 13 with and n = 14 without tachypnoea), and HFNCcpap (n = 14 with and n = 15 without tachypnoea). On postnatal days 13-14: control (n = 12), nCPAP (n = 9 with and n = 11 without tachypnoea), HFNC (n = 11), and HFNCcpap (n = 11).

**Figure S1: Custom-tailored plastic nasal mask for newborn lambs**


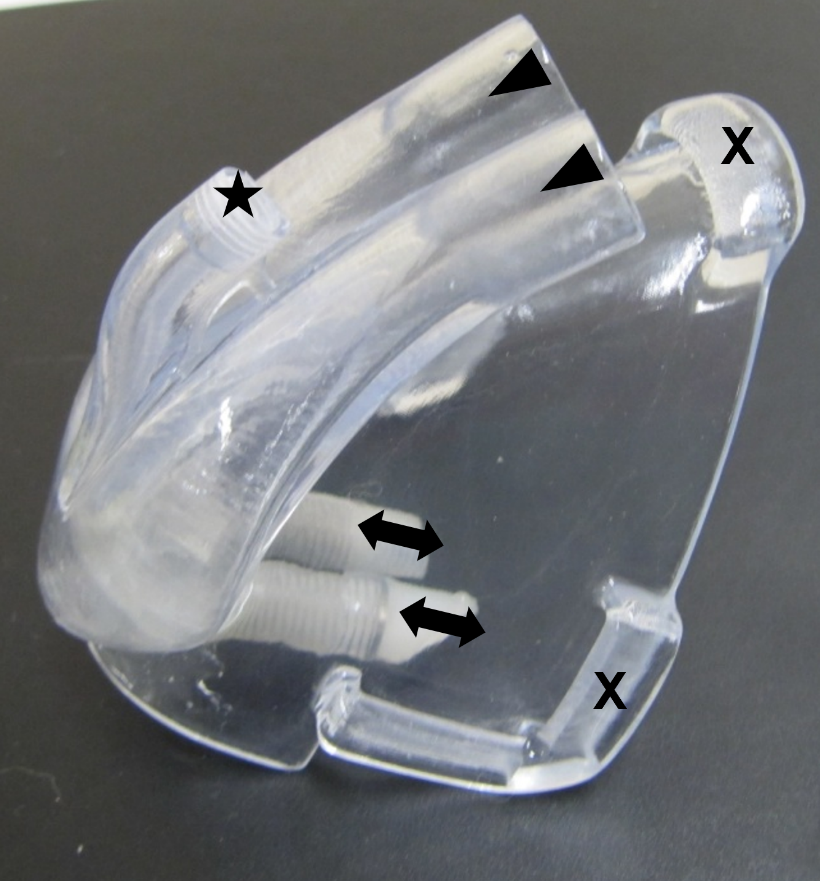


The nasal mask was designed in collaboration with the Department of Mechanical Engineering of the University of Sherbrooke. The nasal mask does not impinge on the mouth and is secured with headgear. It also includes two short nasal cannulas. The inner part of the mask is filled with dental paste to decrease the dead space and to prevent leaks.

Double arrows: nasal cannulas; arrowheads: connectors to the Infant Flow device; star: mask pressure port; X: attachment points for the headgear.

**Figure S2: Custom-designed bottle-feeding device** (Alain et al., 2021)

**
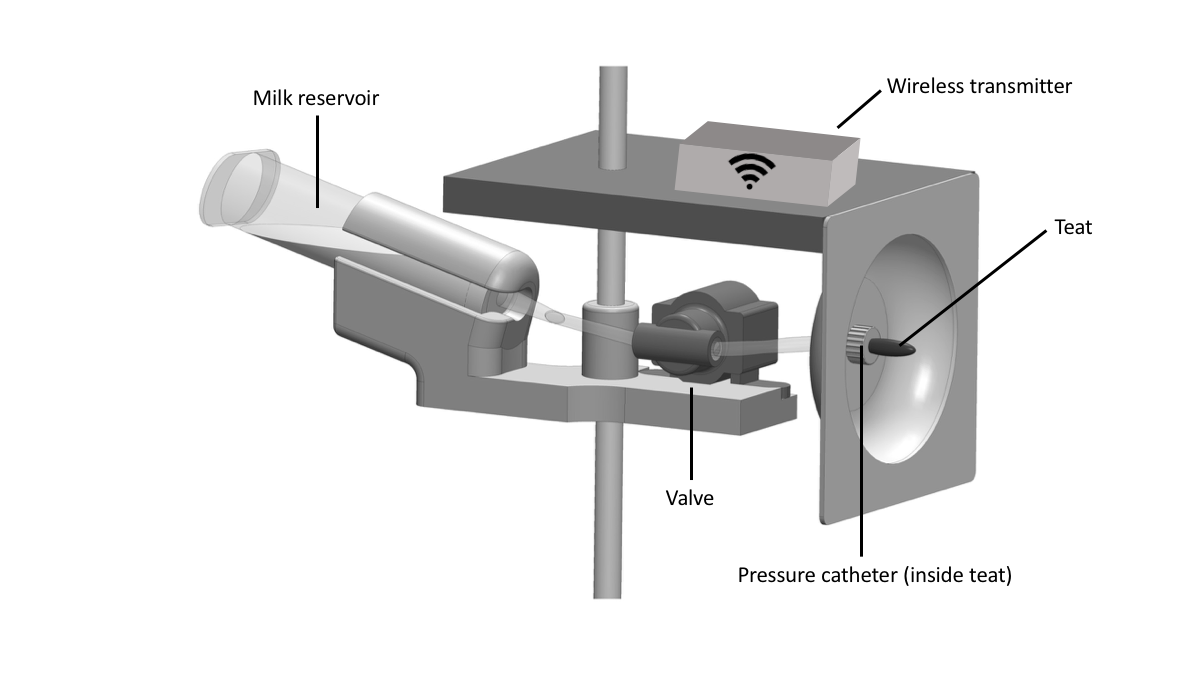
**

This custom-designed bottle-feeding device allowed us to standardize the volume of milk given, as well as to record sucking via a pressure catheter inside the teat. The pressure catheter was connected to a wireless transmitter that relayed the sucking pressure to a computer.
